# Supplementary material for: Adding four-dimensional data assimilation by analysis nudging to the Model for Prediction Across Scales – Atmosphere (version 4.0)
Source: Geosci Model Dev. Author manuscript; Available in PMC 2019 Apr 22. (PMC6475925; doi:10.5194/gmd-11-2897-2018)
Supplement: Supp Info [file NIHMS1013853-supplement-Supp_Info.zip › gmd-11-2897-2018-supplement-title-page.pdf]

Supplement of Geosci. Model Dev., 11, 2897–2922, 2018
https://doi.org/10.5194/gmd-11-2897-2018-supplement
© Author(s) 2018. This work is distributed under
the Creative Commons Attribution 4.0 License.

Geoscientific Model Development
Open Access

Supplement of

Adding four-dimensional data assimilation by analysis nudging to the Model for Prediction Across Scales – Atmosphere (version 4.0)

Orren Russell Bullock Jr. et al.

Correspondence to: Orren Russell Bullock Jr. (bullock.russell@epa.gov)

- gmd-11-2897-2018-supplement-title-page.pdf
- Model_code_for_MPAS-A_with_FDDA
  - MPAS Copyright.txt
  - MPAS-Release-4.0_plusFDDA.tar.gz
  - README.txt
- scripts_for_FDDA_file_preparation
  - prepare_FDDA_file_Jan2013.csh
  - prepare_FDDA_file_Jul2013.csh
  - prepare_FDDA_file_README.txt

The copyright of individual parts of the supplement might differ from the CC BY 4.0 License.
